# Thyroid Function Alteration in Obesity and the Effect of Bariatric Surgery

**DOI:** 10.3390/jcm11051340

**Published:** 2022-02-28

**Authors:** María Cordido, Paula Juiz-Valiña, Paula Urones, Susana Sangiao-Alvarellos, Fernando Cordido

**Affiliations:** 1 Grupo Fisiopatoloxía Endócrina, Nutricional e Médica (FENM), Facultad de Ciencias de la Salud, Universidade da Coruña, 15006 A Coruña, Spain; maria.cordido.carro@sergas.es (M.C.); paula.juiz.valina@sergas.es (P.J.-V.); paula.urones.cuesta@sergas.es (P.U.); 2 Instituto de Investigación Biomedica (INIBIC), Centro de Investigaciones Científicas Avanzadas (CICA), Universidade da Coruña, 15006 A Coruña, Spain; 3 Servicio Urgencias, Hospital Universitario A Coruña, 15006 A Coruña, Spain; 4 Servicio Endocrinología y Nutrición, Hospital Universitario A Coruña, 15006 A Coruña, Spain

**Keywords:** obesity, endocrine abnormalities, bariatric surgery, hypothyroidism

## Abstract

The most common endocrine disease in obesity is hypothyroidism and secondary endocrine alterations, including abnormal thyroid function, are frequent in obesity. It is unclear whether impaired thyroid function is the cause or the consequence of increased adiposity; furthermore, there are no clear data regarding the best way to dose levothyroxine for patients with both hypothyroidism and obesity, and the effect of bariatric surgery (BS). The aim of the present article is to review some controversial aspects of the relation between obesity and the thyroid: (1) Thyroid function in obesity and the effect of BS (2) Thyroid hormone treatment (THT) in obese patients with hypothyroidism and the effect of BS. In summary: In morbidly obese patients, TSH is moderately increased. Morbid obesity has a mild central resistance to the thyroid hormone, reversible with weight loss. In morbidly obese hypothyroid patients, following weight loss, the levothyroxine dose/kg of ideal weight did not change, albeit there was an increment in the levothyroxine dose/kg of actual weight. From a clinical practice perspective, in morbid obesity, diagnosing mild hypothyroidism is difficult, BS improves the altered thyroid function and THT can be adapted better if it is based on ideal weight.

## 1. Introduction

The thyroid hormone (TH) controls dietary intake as well as energy expenditure, both resting and total, and consequently, obesity and different metabolic diseases can appear in patients with altered thyroid function. Furthermore, altered thyroid function is characterized by the presence of changes in total body weight and total body composition, body temperature, and metabolic expenditure [1].

Thyroid function studies are frequently indicated in the evaluation of obesity etiology [2]. It is common to find slightly increased values of thyrotropin (TSH) in obesity [3,4]. It is unclear whether the altered thyroid function present in obesity is due to the excess adiposity or, alternatively, the decreased thyroid function is the cause of the excess adiposity. The thyroid axis regulates the adipose tissue and the adipose tissue affects the activity of the thyroid axis [5].

Obesity could be considered a disease of the nervous system [6] and is a great problem to the health system at the present time, with very important consequences for health care and society [7]. In recent years, the number of obese patients has progressively augmented. In the last 40 years, obesity has reached epidemic proportions and obesity-related diseases have consistently increased in the last 30 years due, predominantly, to cardiovascular disease [8]. Spain has a prevalence of obesity of 22.9% [9] and in almost all European countries is more than 20% [2]. The age-adjusted prevalence of obesity in the USA is 40.4% in women and 35.0% in men. The corresponding values for class 3 obesity (with BMI ≥ 40 kg/m^2^) are 9.9% for women and 5.5% for men [10]. A projection study indicates that the prevalence in the USA of obesity in adults and obesity with a BMI ≥ 35 kg/m^2^ will increase [11]. Slightly lower results have been found all over the world [12]. As little as 5% weight loss improves the function in different organs and tissues concurrently, and progressively increased weight decrement induces changes in key adipose tissue biological pathways [13,14]. Bariatric surgery (BS), using laparoscopic banding, sleeve gastrectomy (SG), or a laparoscopic Roux-en-Y Gastric Bypass (RYGB), compared with medical treatment, has produced more marked ameliorations in diseases associated with obesity and a more marked decrease in all-cause mortality [15]. In obesity, bariatric surgery was associated with longer life duration than medical treatment, even though mortality remained elevated, in both the surgical and usual care of obese groups when compared with the general population [16]. A clear amelioration in obesity-related diseases has also been found following BS. These benefits in patients occur early, before the presence of any significant weight loss, so that benefits may be probably due to the gastrointestinal hormonal secretion modifications due to bariatric surgery [17]. In marked contrast, in patients with obesity and Type 2 diabetes treated with RYGB surgery or diet, the clinical improvement of RYGB surgery and diet were very similar and were apparently related to weight loss itself, suggesting that the benefits of BS are exclusively due to the effect of weight loss [18].

The most prevalent endocrine disease in obesity is subclinical primary hypothyroidism. It is suggested to test all patients with obesity for the presence of altered thyroid function [2]. Hypothyroidism, defined as an increased circulating TSH value, affects up to 10% of the adults, affecting more women than men [19]. Obesity is accompanied with endocrine alterations, including a decreased growth hormone (GH) response to different stimuli [20,21,22,23,24] and altered thyroid function [22,25]. Thyroxine values have been found to be normal, increased, and decreased in obesity; these different results are likely due to the fact that the patients were examined at various time periods, have different sexes and ages, and may differ in the severity and kind of obesity as well as in the presence of obesity comorbidities [3,4,25,26,27,28]. The disturbance of thyroid function in obese patients and the role of BS treatment on thyroid function change is unclear at the present time. There are different studies showing various results in connection with the change of thyrotropin after BS and the influence of weight decrement [28,29,30,31,32,33,34]. The free thyroxine (FT4) results in obese patients and the influence of BS are even more controversial [29].

As previously mentioned, the most prevalent endocrine disease in obesity is decreased thyroid function and it is recommended to measure circulating thyroid hormones in all obese patients [2]. In clinical practice, body weight is frequently used to estimate the total dose of levothyroxine (LT4) to administer in the presence of decreased thyroid function [35]. There are various reasons for increased requirements of levothyroxine in obese subjects: increased lean and fat mass [36], increased volume of distribution, or altered gastrointestinal tract absorption [37]. As such, weight loss from BS may reduce the levothyroxine needs [38,39]. Conversely, the surgical technique, by altering the anatomy and physiology of the gastrointestinal tract, could induce a decrease in the absorption of the hormone and, therefore, increase the requirements of levothyroxine [40].

The objective of the present article is to review some controversial aspects of the relation between obesity and thyroid function: (1) thyroid function in obesity and the influence of bariatric surgery; (2) thyroid hormone replacement in obese patients with hypothyroidism and the influence of bariatric surgery.

## 2. Thyroid Function in Obesity and the Effect of Bariatric Surgery

TH values have been found to be decreased, increased, and normal in obesity [3,4,26,28]. Our group has found increased TSH values in morbidly obese patients [34]. Rotondi et al. [4] have found augmented circulating TSH values in an ample group of severely obese subjects when compared with healthy normal weight subjects. Reinehr et al. [27] reported increased thyrotropin levels in obese children when compared with normal weight children. The degree of overweight correlated with circulating thyrotropin results. Valdes et al. [3] have found augmented circulating TSH values in severe obesity, and have suggested that reference values for thyrotropin may be unsuitable to define decreased thyroid function in persons who are severely obese.

At present, it is unclear if the slight elevation of TSH values present in obesity causes weight gain or if it is due to obesity induced activation of the hypothalamus-pituitary-thyroid (HPT) axis, causing an increase in serum TSH. In support of the latter theory, there are studies that have found that thyrotropin values decrease following surgery [34,41]. In order to clear up this question, Wang et al. [42] employed data from genome wide association studies to carry out a bidirectional mendelian randomization analysis. They found that inverse variance-weighted and mendelian randomization-Egger results indicated that genetically driven circulating thyrotropin did not lead to changes in the body mass index or increased body weight. Additionally, the inverse variance-weighted method showed that the circulating thyrotropin values could be increased by a genetically predicted high body mass index. These data clearly suggest that obesity can significantly increase TSH [42]. In addition, if increased TSH is considered a consequence of increased adiposity, our clinical practice should be modified. Slight increased thyrotropin in obese patients without any other data of decreased thyroid function should be considered normal. Nevertheless, increased thyrotropin circulating levels may elicit detrimental effects due to its actions in extra thyroidal tissues [43].

The consequences and mechanisms of increased circulating TSH in obese patients are unclear. Different mechanisms could explain the relationship of decreased thyroid function to increased adipose tissue [44]. There is a direct role of circulating thyrotropin levels in the physiological regulation of thermogenesis [45]. Thyrotropin receptors are decreased in obese patients when compared with normal subjects [46]. The elevation of circulating thyrotropin levels may be due to a compensatory activation of the HPT axis in response to increased adiposity [26]. In accordance with that hypothesis, in morbid obesity after weight loss, a positive relationship between resting energy expenditure and free thyroxine (FT4) has been found [47] and elevated FT4 has been found in obese patients [27]. This activation could be mediated by central actions of the adipose tissue hormone leptin [48]. In contrast, Marzullo [49] et al. have found that increased adiposity could trigger autoimmunity against the thyroid, suggesting that increased adiposity could be a casual mechanism for established thyroid disease.

The effect of bariatric surgery on postoperative thyroid activity evolution remains incompletely understood. Several studies have found different results regarding the variation in circulating thyrotropin values following BS and the relation of thyrotropin variation with weight decrement [28,29,30,31,32,33]. Most [29,30,32], but not all [28,33], studies have found a decrease of circulating thyrotropin values post-intervention. Our group carried out a study evaluating 129 euthyroid patients with morbid obesity before and after BS. Thyrotropin declined over time, and the thyrotropin reduction was associated with the excessive BMI loss [34]. Similar results have been found in the systematic review and meta-analysis from Guan et al. [29]. Neves et al. [32] evaluated euthyroid obese subjects and found that bariatric surgery induces a decrement of thyrotropin levels and that the decrease was associated with the weight loss following BS. In contrast, Dall’Asta et al. [33] performed an observational study evaluating healthy subjects and obese patients after gastric banding induced weight loss and found that thyrotropin values did not change. Zhang et al. [28] followed and evaluated obese subjects after RYGB surgery and found that thyrotropin values remained stable. In the study of Guan et al., free thyroxine did not change following BS [29]. According to their data, in obese patients after BS, no variation was found in free thyroxine or free triiodothyronine values [50].

The mechanisms of the thyrotropin decrease after bariatric surgery are not yet fully understood. This fall of circulating thyrotropin levels is weight loss mediated and is not due to an effect of bariatric surgery. A decrease of circulating thyrotropin has been found in obesity after lifestyle change caused weight loss [51], and the decrement of the thyrotropin values was associated with excessive weight loss following bariatric surgery [32,34], suggesting that the decrease in thyrotropin is primarily due to the weight loss. The decrease of circulating leptin values after bariatric surgery could be the mechanism responsible for the decreased stimulation of the hypothalamus-pituitary-thyroid axis [48,52] and the decline of circulating thyrotropin values. Besides, reduced thyrotropin receptor expression and thyrotropin resistance is improved by weight loss [46]. Moreover, due to the link between the hypothalamus-pituitary-IGF-I axis and excessive adiposity [20,21,22,53,54] and the influence of GH on the thyroid axis [55], the relationship between the hypothalamus-pituitary-IGF-I axis and thyrotropin has been studied, but with negative results [34].

From a clinical perspective, the increased thyrotropin values in obesity stand out that it is an important clinical problem to diagnose the presence of mild hypothyroidism in obesity [25]. Hypothyroidism should be considered in obesity with marginally elevated thyrotropin values only after measuring circulating levels of TH and thyroid autoantibodies, and with data suggesting decreased TH action [25]. Marginally elevated circulating thyrotropin values could be due to a compensatory response to morbid obesity and not a real decreased thyroid hormone action [3]. Furthermore, these data do not suggest the diminution of the upper limit of thyrotropin values [56,57] to diagnose hypothyroidism in obese patients.

Elevated free thyroxine coexist with elevated thyrotropin in the clinical situation of resistance to TH, an inherited rare disease [58,59]. There are two types of resistance to thyroid hormone, hypothalamus-pituitary resistance, and decrease peripheral resistance to TH’s actions. The pituitary resistance can be assessed measuring circulating TH values and TSH values or more precisely with the thyrotroph T4 Resistance Index (TT4RI) and TSH index (TSHRI) [60,61]. In extremely obese patients, TH values and thyrotropin values tend to be elevated [62]. Hence, the increased thyroxine and high thyrotropin can be conciliated if increased thyroxine and increased thyrotropin circulating values are due to a central resistance to TH. Further, the resistance could be also present at a peripheral level. An acquired mild resistance to thyroxine has been suggested by Tjorve et al. [63]. Laclaustra et al. [64] have found a clear relation between central resistance to the thyroid hormone and the prevalence of metabolic diseases like obesity and diabetes in a representative sample of the population of the United States of America.

TH action is different for each tissue, depending on TH values and on the unique mixture of cell membrane transporters, deiodinases, and thyroid hormone receptors present in each tissue [65]. The resistance to TH indices measure pituitary resistance, the thyrotropin inhibition by circulating free thyroxine values. Moreover, the presence of obesity suggests decreased thyroid function and supports that peripheral resistance to the thyroid hormone is also present [64]. Additionally, there are experimental models of reduced thyroid hormone action in excessive adiposity [65,66]. Thyroid hormone receptors are diminished on the adipose tissue of obese patients [46]. Moreover, the thyroid hormone receptor β has been found to be related with disease severity in liver tissue from morbidly obese patients with different phases of hepatic injury, following BS [67]. Furthermore, sensitivity to the thyroid hormone could be epigenetically regulated [68]. Our group has found elevated TT4RI and TSHRI in obese patients and these indices decline with weight loss [69], suggesting that the elevation of circulating thyrotropin values and free thyroxine levels could be due to a stimulation of the HPT axis [26].

The mechanisms that explain bariatric surgery induced diminution in the indices of central resistance to the thyroid hormone are unclear. The decline in the indices of central resistance to thyroid hormone has been found related to excessive weight loss following bariatric surgery [69]. This decline is likely due to weight loss itself. A diminution in circulating thyrotropin levels has been found in obesity following lifestyle induced weight loss [51] and the decline in thyrotropin levels has been found associated with excessive weight loss after bariatric surgery [32,34]. According to these data, TH resistance improves due to an increase in the reduced thyroid hormone receptor expression with weight loss [46]. The decrease in circulating leptin values [52], due to the decrease in body fat, could reduce the pituitary stimulation of the thyroid axis [48] and induce a decrease of thyrotropin. Nevertheless, leptin treatment did not modify the thyroid anatomy [70]. In the presence of extreme resistance to insulin action, the prevalence of thyroid nodules and the goiter is increased [70]. Conversely, Juiz-Valiña et al. could not find any relationship between insulin resistance and TT4RI or TSHRI [69]. Growth Hormone secretion is altered in obese patients [20,21,22,53,54], and circulating GH values could regulate the HPT axis. Growth hormones increased, circulating free triiodothyronine, and diminished, circulating free thyroxine values [55]. Growth hormone treatment in adult patients causes different changes in the thyroid axis, the most important is decreased free thyroxine values [71,72]. In obesity, there is a markedly decreased GH secretion, this alteration could be responsible of the increased free thyroxine values. Juiz-Valiña et al. found no relationship between the GH-IGF-1 axis and the central resistance to TH [69]. In contrast, indices of inflammation, such as c-reactive protein, highly correlate with TT4RI or TSHRI in obesity [69]. In accordance with those results, SG induces thyrotropin diminution in obesity, which correlates with an improved inflammatory state after BS [73]. In summary, the results of Juiz-Valiña et al. suggest that the decrease in TT4RI or TSHRI following bariatric surgery is primarily due to weight loss and the improved inflammatory state could be a contributory factor [69]. These data also suggest that the improvement of thyroid function is another benefit of bariatric surgery [74]. Recently reduced pituitary sensitivity to TH has been found to be associated with diabetes and hypertension in a representative group of Iranian euthyroid subjects [75]. These data reinforce the importance of the relation between reduced central sensitivity to thyroid hormone with metabolic diseases. The most important aspects of thyroid function in obesity and the effect of bariatric surgery are summarized in Table 1.

## 3. Thyroid Hormone Treatment in Obese Patients with Hypothyroidism and the Effect of Bariatric Surgery

Actually, it is not clear what happens to the dose of thyroxine in hypothyroid patients after bariatric surgery. Thus, there is a clear need of additional studies [76]. Furthermore, the effect of distinct bariatric surgery techniques on levothyroxine absorption is not clear [77]. Ojomo et al. [35] have studied thyroid hormone replacement after thyroidectomy in 122 patients and they conclude that the present standard of weight-based thyroid hormone replacement (THR) does not adequately dose underweight and overweight patients [35]. The most common starting dose of levothyroxine after a total thyroidectomy is 1.6 µg/kg [78]. This dose is adjusted based on circulating thyrotropin levels and clinical data. The dosage recommendations are often ambiguous, regarding whether the dose is based on the current total body weight (BW), estimated ideal BW (IBW) or estimated lean body weight (LBW). Obese subjects can develop hyperthyroidism if dosing is based on the same data used for non-obese subjects [79]. A more adequate thyroid hormone replacement should include the BW and body mass index of the obese patient [78].

The altered thyroxine absorption induced by bariatric surgery has been analyzed, albeit with questionable results. Since the introduction of obesity surgery in clinical practice, doubts about altered drug absorption have been featured [80,81]. Decreased thyroid function treatment mandates oral THR, and there are concerns about its adequacy after bariatric surgery [36]. The primary thyroxine absorption site is the small intestine in the jejunum and the ileum [37]. Various endogenous and exogenous factors may disturb intestinal levothyroxine absorption [82]. Bariatric surgery could probably be included in the list of factors that alter intestinal levothyroxine absorption [76]. Surgery procedures with gastric restriction (gastric banding and sleeve gastrectomy), are procedures that modify drug absorption less than procedures involving intestinal diversion [83]. Levothyroxine absorption has been found similar before and after RYGB [36]. Different studies have found an increased need for levothyroxine after jejunoileal bypass techniques [84,85,86]. These data are in keeping with the increased thyrotropin levels found in patients treated with the same levothyroxine dose after bariatric surgery [87,88]. Furthermore, others have found a diminution in thyroxine needs after BS [38,89]. Rudnicki et al. [90] have described that bariatric surgery improved thyroid function in hypothyroidism. Similar improvements in levothyroxine doses after bariatric surgery have been found by other authors [89,91,92]. A recent meta-analysis [41] has found that bariatric surgery promotes a decrease in the total levothyroxine requirement. In the study of Pedro et al. [77], the total levothyroxine dose before and 12 months following bariatric surgery was similar, however, the thyroxine dose/kg of actual weight was increased. Other authors have found similar results with a decrease or no change in the total levothyroxine requirement, but an increase of the weight-based levothyroxine requirement after bariatric surgery [93]. Our group has encountered that, following bariatric surgery, the total levothyroxine or the levothyroxine dose/kg of IBW did not change; however, the levothyroxine dose/body surface area, levothyroxine dose/kg of actual BW, levothyroxine dose/kg of adjusted BW and levothyroxine dose/kg of LBW increased [94].

It has been encountered that the weight-based daily levothyroxine dose increased following a Roux-en-Y Gastric Bypass, with no significant changes after sleeve gastrectomy [95]. These result highly suggest that sleeve gastrectomy and a Roux-en-Y Gastric Bypass showed different changes in levothyroxine needs. However, in agreement with Pedro et al. [77], our group study did not find the bariatric surgery technique to be a predictor of levothyroxine dose variation [94]. Similar results have been found by other authors; both sleeve gastrectomy and a Roux-en-Y Gastric Bypass improved thyroid function in the same way [90]. These data support that both procedures have similar effects on levothyroxine absorption. Altered gastric emptying modifies the levothyroxine absorption [96], and altered gastric emptying is present in the Roux-en-Y Gastric Bypass and sleeve gastrectomy [97]. This could be the mechanism that explains the similar results between the malabsorptive and restrictive techniques.

The mechanisms for bariatric surgery levothyroxine dose variations are unclear. Rubio et al. [36] have found a delay of levothyroxine absorption in the surgically-treated patients. Gkotsina et al. [40] have encountered that the pharmacokinetic data were similar following a Roux-en-Y Gastric Bypass, and that levothyroxine pharmacokinetics improve after a biliopancreatic diversion [40]. Conversely, has been found that by employing the same dose of levothyroxine before and after bariatric surgery, the serum TSH increased following bariatric surgery [88]. Levothyroxine needs are increased with malabsorptive procedures, and results about thyroxine needs with procedures combining restrictive and malabsorptive techniques are conflicting. This could be due to the different schedule of levothyroxine ingestion, the diverse effects of bariatric surgery and other endogenous and exogenous factors, like other drugs administration [97,98]. In obese, diabetic patients with primary hypothyroidism and treated with levothyroxine, metformin treatment provokes a decrease in circulating TSH [99]. Most of the studies have found that bariatric surgery induces a decrease in total levothyroxine dose [41]. Nevertheless, the different characteristics of the studies does not make it possible to draw definitive conclusions about the net effect on levothyroxine needs [100]. Furthermore, the evaluation of levothyroxine dosing is not always adjusted for weight and so does not allow for a correct comparison of the data. Lean body mass modification after bariatric surgery could also contribute to the change in levothyroxine doses [79]. Rudnicki et al. [90] have found a decrease of levothyroxine dosage after bariatric surgery in hypothyroid patients. Moreover, impaired levothyroxine pharmacokinetics in obese patients have been indicated [93]. In summary, in most of the studies, weight loss following bariatric surgery provokes a diminution in total levothyroxine needs. Juiz-Valiña et al. have found that in obese hypothyroid patients treated with BS, the absolute levothyroxine dose did not change, nor did the levothyroxine dose/kg of IBW, but the levothyroxine dose/body surface area, levothyroxine dose/kg of actual BW, or levothyroxine dose/kg of LBW significantly increased [94]. A change in the absolute levothyroxine dose and levothyroxine dose/kg of IBW was not related to excessive weight loss. On the contrary, excessive weight loss was related to an increase in the levothyroxine dose/body surface area, and levothyroxine dose/kg of present BW [94]. These data suggest that the thyroid hormone replacement change after bariatric surgery is due to a mixed mechanism, a decrease in levothyroxine needs due to weight loss and a decrease in levothyroxine absorption due to the surgical procedure. Decreased thyroid function treated patients could be metabolically different when compared with normal subjects. Accordingly, it has been found that obese hypothyroid women on levothyroxine therapy, with normal circulating thyrotropin values, have a diminished energy expenditure, suggesting that standard levothyroxine replacement does not fully correct metabolic alterations related to hypothyroidism [101]. Furthermore, other studies have encountered that hypothyroid patients treated with levothyroxine showed higher adiposity and similar insulin resistance, but healthier lipid levels compared with euthyroid obese patients [102].

The best way to titrate the levothyroxine dose for patients with decreased thyroid function and increased adiposity is not clear [78]. A weight-based dosing of the thyroid hormone inappropriately overdoses obese patients [78]. A better way for thyroid hormone dosing could consider other aspects, such as both the weight and BMI of the obese patient, and recommended using either the present weight of the obese subject with adjustment of the dose, considering the BMI or the adjusted BW [78]. Other authors have considered that the best way to titrate the levothyroxine dose is taking into account lean body mass [79]. In obese patients with a diminished thyroid function, the demand for levothyroxine is increased due to augmented fat mass and lean body mass [30]. Furthermore, in a multivariable analyses study, the levothyroxine dose was predicted by the amount of fat-free mass, hypothyroidism etiology, and the sex of the patients [102]. Juiz-Valiña et al. observed that in extreme obese patients, after bariatric surgery the levothyroxine dose/kg of IBW did not change, however, the levothyroxine dose/body surface area, levothyroxine dose/kg of actual BW, or levothyroxine dose/kg of lean BW increased [94]. In summary, thyroid hormone replacement titration in hypothyroid patients with excessive adiposity can be adjusted more correctly based on IBW. The most important aspects of thyroid hormone treatment in obese patients with hypothyroidism and the effects of bariatric surgery are summarized in Table 2.

## 4. Conclusions and Clinical Implications

In morbidly obese patients, TSH is moderately increased (Table 3). Weight loss provokes a diminution of the elevated thyrotropin values. This decrease of thyrotropin after BS is dependent on the excessive weight lost. These data suggest that the moderately elevated thyrotropin values present in obese patients are due to the increased adiposity of obesity. From a clinical practice point of view, diagnosing mild hypothyroidism is difficult in severe obesity, and BS improves the mild hypothyroidism of severe excessive adiposity (Table 4).

Morbid obesity is characterized by a mild reversible pituitary resistance to the thyroid hormone. Weight loss induced with bariatric surgery causes a reduction in the increased pituitary resistance to TH.

In morbid hypothyroid obese patients, following weight loss, the total levothyroxine dose decreased and the levothyroxine dose/kg of IBW did not change in most of the studies. However, the levothyroxine dose/kg of actual BW increased.

In morbid hypothyroid obese patients, after BS, the diminution in the percentage of weight lost was significantly inversely correlated with the levothyroxine dose/body surface and the levothyroxine dose/kg of actual BW. Additionally, the absolute levothyroxine dose and the levothyroxine dose/kg of IBW was not related with weight loss.

The levothyroxine needs and its change after bariatric surgery was similar for Sleeve Gastrectomy and Roux-en-Y-Gastric Bypass.

From a clinical practice perspective, thyroid hormone replacement in patients with obesity and hypothyroidism can be more adequately adjusted if it is based on IBW, and RYGB does not affect levothyroxine absorption differently from SG.

## Figures and Tables

**Table 1:** Thyroid function in obesity and the effect of bariatric surgery.

Thyroid Function in Obesity and the Effect of Bariatric Surgery
- In severe obesity, thyrotropin is moderately increased. The slightly elevated thyrotropin in obese patients is due to increased adiposity.
- Weight loss provokes a diminution of the elevated thyrotropin values. The decrease of thyrotropin after BS is dependent on the excessive weight loss.
- BS improves the subclinical hypothyroidism of morbid obesity.
- Morbid obesity is characterized by a mild central resistance to the thyroid hormone. Weight loss induced with BS cause a reduction in the increased pituitary resistance to thyroid hormone.

BS, Bariatric Surgery.

**Table 2:** Thyroid hormone treatment in obese patients with hypothyroidism and the effects of bariatric surgery.

Thyroid Hormone Treatment in Obese Patients with Hypothyroidism and the Effects of Bariatric Surgery
- In hypothyroid severe obese patients following BS-induced weight loss, the total levothyroxine dose decreased, the levothyroxine dose/kg of IBW did not change and the levothyroxine dose/kg of actual BW increased.
- In hypothyroid severe obese patients, after BS, the weight lost was inversely correlated with the levothyroxine dose/body surface and levothyroxine dose/kg of actual BW. The absolute levothyroxine dose and the levothyroxine dose/kg of IBW was not related with weight loss
- The levothyroxine needs and its change after BS was similar for SG and RYGB
- Thyroid hormone replacement in patients with obesity and hypothyroidism can be more adequately adjusted if it is based on IBW.

BW, body weight; IBW, ideal body weight; BS, Bariatric Surgery; RYGB, Roux-en-Y-Gastric Bypass; SG, Sleeve Gastrectomy.

**Table 3:** Essential points.

Essential Points
- In morbid obese patients, thyrotropin is moderately increased.
- The slightly elevated thyrotropin encountered in obese patients is reversible with weight loss and due to the increased adiposity.
- Morbid obesity is characterized by a slight pituitary resistance to thyroid hormones that is reversible with BS-induced weight loss
- In hypothyroid patients treated with levothyroxine and with obesity, following BS-induced weight loss, the total levothyroxine dose decrease, the levothyroxine dose/kg of actual weight increase and the levothyroxine dose/kg of IBW was stable, in most of the studies.

IBW, ideal body weight; BS, Bariatric Surgery.

**Table 4:** Clinical implications.

Clinical Implications
- Clinically, the diagnosis of subclinical hypothyroidism is difficult in severe obesity, and BS improves the mild subclinical hypothyroidism present in severe obesity.
- The levothyroxine needs following BS were similar for SG and RYGB.
- From a clinical practice perspective, thyroid hormone replacement in patients with obesity and hypothyroidism can be more adequately adjusted if it is based on IBW.

IBW, ideal body weight; BS, Bariatric Surgery; RYGB, Roux-en-Y-Gastric Bypass; SG, Sleeve Gastrectomy.

## Data Availability

Not applicable.
